# A study of the relationship between the level of anxiety declared by MRI patients in the STAI questionnaire and their respiratory rate acquired by a fibre-optic sensor system

**DOI:** 10.1038/s41598-019-40737-w

**Published:** 2019-03-13

**Authors:** Łukasz Dziuda, Piotr Zieliński, Paulina Baran, Mariusz Krej, Lech Kopka

**Affiliations:** 1 Department of Flight Simulator Innovations, Military Institute of Aviation Medicine, ul. Krasińskiego 54/56, 01-755 Warszawa, Poland; 2 Department of Aviation Psychology, Military Institute of Aviation Medicine, ul. Krasińskiego 54/56, 01-755 Warszawa, Poland; 3 Centre of Experimental Medicine, Military Institute of Aviation Medicine, ul. Krasińskiego 54/56, 01-755 Warszawa, Poland

## Abstract

Magnetic resonance imaging (MRI) patients often experience anxiety-related respiratory disorders, including hyperventilation, but their respiratory indicators are not routinely monitored during scanning. Free from metal parts and immune to electromagnetic radiation, fibre-optic sensors have the potential to better control the patient’s condition by providing continuous non-invasive monitoring of the respiratory rate (RR). The study was purposed to assess the relationship between anxiety in MRI patients and their RR acquired by a fibre-optic sensor system. Forty-four subjects were involved in the study. The mean RR values recorded for 2 minutes immediately after the beginning and immediately before the end of the scanning were assessed relative to the State-Trait Anxiety Inventory (STAI) X-1 scores obtained immediately before and immediately after the scanning, respectively. A growth mixture model analysis was performed to statistically differentiate two groups of subjects according to the trends in repeated measures of RR. A significant lowering of the anxiety state was observed in the group characterised by a decrease in RR, whereas essentially no change in anxiety level was observed in the group with a stable RR. The *t*-test showed significant differences in changes in anxiety between these groups (*t*_(39)_ = −2.349, *p* = 0.012, Cohen’s *d* = 2.13).

## Introduction

Along with the increasing prevalence of diagnostics with the use of magnetic resonance imaging (MRI), the problem of anxiety in MRI patients and other claustrophobia-related symptoms has become more and more pronounced^1,2^. The main source of anxiety is the need to remain lying down in the narrow gantry space during the examination. Other factors such as a concern about the examination result, high noise, vibration, isolation from the environment, or immobilisation of the head during brain scans may further increase the level of anxiety in the patient. For these reasons, the reported incidence of premature termination or failure of the MRI examination ranges between 0.5% and 14.5%, and the reported incidence of anxiety‐related reactions during MRI reaches 37%^3,4^.

Psychologists examine the level of anxiety and mood using various questionnaires, such as the State-Trait Anxiety Inventory (STAI) or the University of Wales Institute of Science and Technology (UWIST) Mood Adjective Checklist (UMACL)^5,6^. However, these methods are contaminated by subjectivity and additionally do not allow the patient to be monitored continuously during the examination itself. Therefore, efforts have been made to connect the current emotional state with indicators that reflect the functioning of the autonomic nervous system (ANS). The literature shows that the ANS and respiratory activity are closely related to emotions such as anxiety or happiness^7,8^. Negative mood states, e.g. anxiety and stress, activate the sympathetic part of the ANS due to widespread depolarization throughout the body, and cause faster and shallower breathing. In light of current research, breathing characteristics have been found to be significant predictors of anxiety^9^. In particular, the data in the literature indicate that an increase in respiratory rate (RR) occurs as a result of an increase in anxiety level^10,11^. In contrast, slow and deep breathing leads to parasympathetic activation because of widespread inhibition and hyperpolarization, and hence a reduction in negative emotional state is achieved^12^. Thus, information on RR provided by an online monitoring system of MRI patients can be crucial to calm the patient through controlled deepening of breath and RR reduction^13^. This, in turn, can lead to a lowering of anxiety to a level sufficient to complete the MRI examination^14^.

In this paper, we present the results of a study on the relationship between anxiety in patients undergoing MRI scanning and their RR as acquired noninvasively by a vital signs fibre-optic sensor system. We determine whether it is possible to identify patients at risk of claustrophobia attack during MRI procedures and if so, we discuss possible ways to enable anxious patients to complete their scans.

## Methods

### Experimental protocol

The experimental protocol complied with the Declaration of Helsinki and was approved by the Ethics Committee of the Military Institute of Aviation Medicine (Decision 01/2014), in which the study was carried out. The study was registered at https://clinicaltrials.gov/ under number NCT03384849. All activities during the study were performed in accordance with the relevant guidelines and regulations. The subjects were informed in detail of the purpose and nature of the study and signed their informed consent for study participation and publication of identifying information/images.

Forty-four MRI patients were involved in the study, including 30 women and 14 men aged 49.8 ± 17.3 (M ± SD) years. Exclusion criteria from the study were the same as exclusion criteria from standard MRI procedures. The part of the body that was being scanned and the time of the MRI examination were not relevant for further analysis. However, we assumed that the study would be no longer than 1 hour, as an average MRI scanning lasts from 20 to 40 minutes and the administration of contrast media might prolong the survey. Before and after the MRI scanning, i.e. according to the repeated measurement scheme, the subjects completed the STAI questionnaire. The remaining part of the study did not differ from a routine MRI examination, except that the sensor mat of the fibre-optic sensor system was located under the subject’s back. The fibre-optic sensor system enabled the MRI operator to monitor the patient’s current RR throughout the whole scan. All the acquired data were automatically archived for further analyses.

### STAI

To measure anxiety in subjects undergoing MRI procedures, the Polish adaptation of the STAI was used^15^. The inventory is designed to measure the level of the current anxiety state in response to a given situation (the X-1 subscale), as well as a relatively stable personal trait (the X-2 subscale). Evaluation of anxiety level in patients was conducted according to a repeated measurement protocol. The subjects completed the STAI X-1 subscale twice, i.e. just before entering the MRI scanner and immediately after leaving the scanner.

### Fibre-optic sensor system

**Figure 1:**
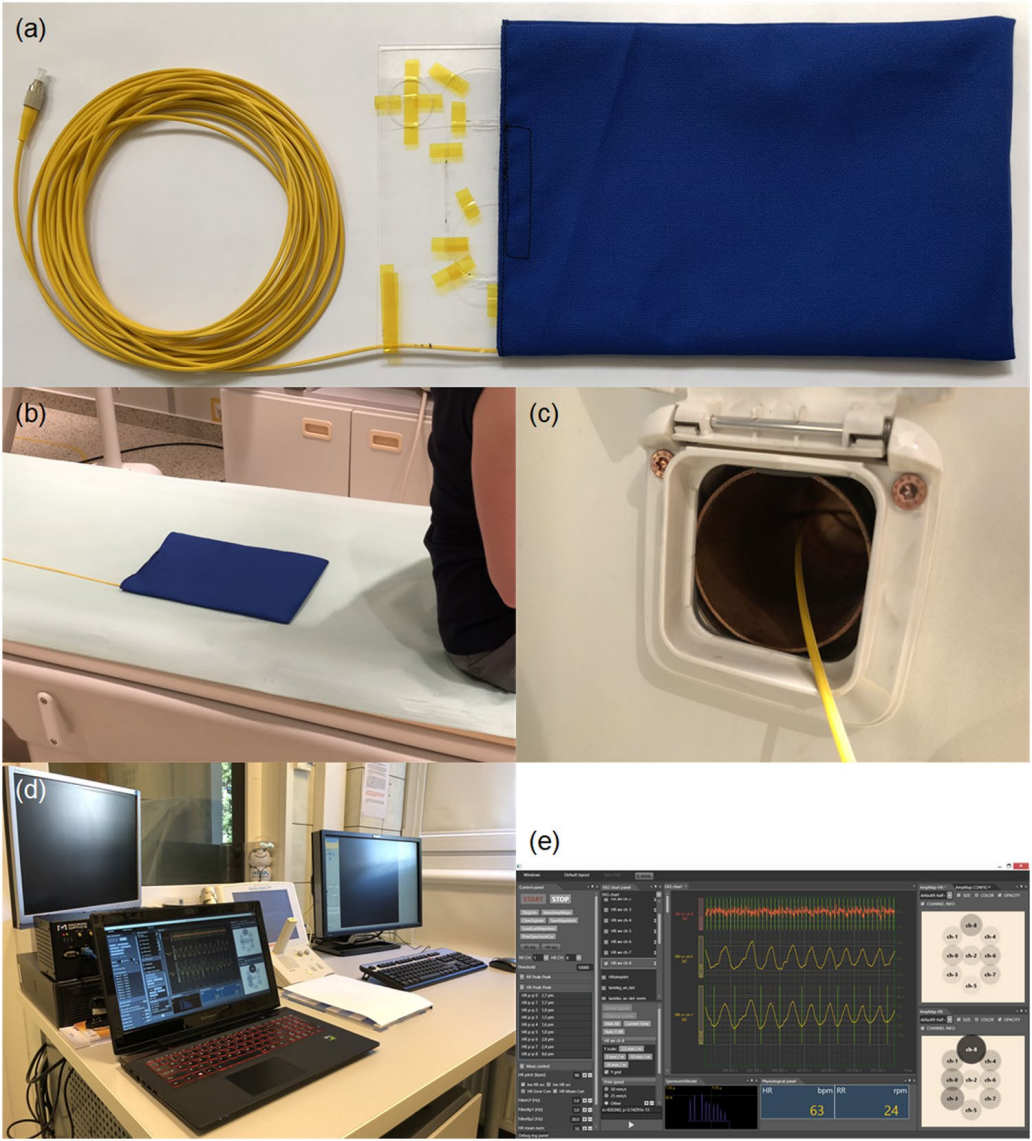
Main elements of the vital signs sensor system: the (**a**) sensor mat with partially exposed spring-board and an FBG element, **(b**) sensor mat on the mattress of the MRI track table, (**c**) optical fibre threaded through the wall opening, (**d**) interrogation module and the laptop PC in the operator room, and (**e**) main window of the application for signal visualisation, RR determination and data archiving.

The sensor system shown in Fig. 1 has been previously laboratory evaluated and implemented into MRI procedures to monitor patient vital signs during the MRI^16,17^. It consists of a sensor mat, an interrogation module and a laptop personal computer (PC). The sensor mat, i.e. the measuring module, includes a spring-board that converts body movements, including lung-induced motions, into strain, and a fibre Bragg grating (FBG) bonded to the board measures this strain (Fig. 1(a)). The instantaneous spectral position of the FBG reflection peak is calibrated in terms of the respiratory readings. The sensor mat was placed in the MRI chamber underneath the chest area of the subject to ensure proximity to the lungs (Fig. 1(b)). A few meters of optical fibre were threaded through a wall opening (Fig. 1(c)) and connected via a fibre-optic for angled physical contact-type connector (FC/APC) to an FBG interrogation module in the operation room. To interrogate the sensor mat, a commercially available sm130–700 integrated unit by *Micron Optics* was used. The data from the interrogation module was sent via an Ethernet interface to the laptop PC (Fig. 1(d)) with software developed for signal visualization, automatic determination of RR and data archiving (Fig. 1(e)).

As mentioned above, the development of the fibre-optic sensor system involved carrying out both the laboratory evaluation and the phase of implementation for the clinical trials. After each laboratory or clinical examination, the subject was asked about the personal experience related to the conducted examination, including a sense of comfort, well-being and feelings associated with the presence of the sensor mat in the MRI chamber. None of the subjects examined so far have reported any critical remarks about the presence of a sensor mat in the MRI chamber during the examination. All the subjects agreed that the sensor mat was practically imperceptible and did not affect their well-being in any way. They have also testified that after taking the place in the MRI scanner they completely forgot about the presence of the sensor mat. In the interviews, the subjects paid particular attention to the noise generated by the MRI scanner during examination. Hence in the current study it was assumed that the fibre-optic sensor system did not affect the anxiety level in the subjects.

### Capnograph

In cases of resignation or interruption of the MRI examination, an in-house-designed capnograph based on the miniMediCO2 module by *Oridion Medical* was used to measure RR just after leaving the MRI room as well as after the subject became calm.

### MRI scanner

A 1.5-Tesla Achieva system by *Philips* was used in the study.

### Statistical analysis

In the first step, the statistical analysis was aimed at checking whether it is possible to predict the risk of the withdrawal of a subject from the MRI examination due to excessive anxiety, based on the STAI X-1 scores obtained before entering the scanner, and based on the mean RR values recorded at the beginning of the scanning. Due to the small sample size, it was predicted that withdrawal occasions will be extremely rare. Therefore, Firth’s logistic regression^18^ with the penalization of log-likelihood was applied to estimate regression models for the STAI X-1 scores and RR values, as it is recommended for avoiding the estimation bias in logistic regression with small sample sizes^19^.

The second step of statistical analysis was conducted on the complete data, i.e. gathered from the subjects that did not withdraw from the MRI examination. It was purposed to check if there were some common trends among the subjects in the change of the anxiety state and RR during MRI examination. At first, descriptive statistical methods were used for the evaluation of the anxiety state and RR indicators as well as for the evaluation of the difference between the first and second anxiety measurement. Then, a correlation analysis with a Pearson product-moment correlation coefficient was performed to check whether the initial RR is related to the anxiety state measured immediately before the MRI examination.

Because we hypothesised that there would be individual variability in the change in RR during the MRI examination, a growth mixture model analysis^20^ was performed to statistically differentiate groups of subjects according to the trends in repeated measures of RR. After that, an analysis with the use of Student’s *t*-test was performed to check whether such identified groups differed on the magnitude of change in state anxiety observed during the study. An alpha level of 0.05 was used for all statistical tests.

All analyses were performed using R statistical software, version 3.5.2, by *R Core Team*. Firth’s logistic regression was performed using the logisf package in version 1.23. The growth mixture model analysis was performed using the lcmm package in version 1.7.7. A power analysis was performed with the pwr package in version 1.2–1, and plots were created using the ggplot2 package in version 2.2.1.

## Results

**Figure 2:**
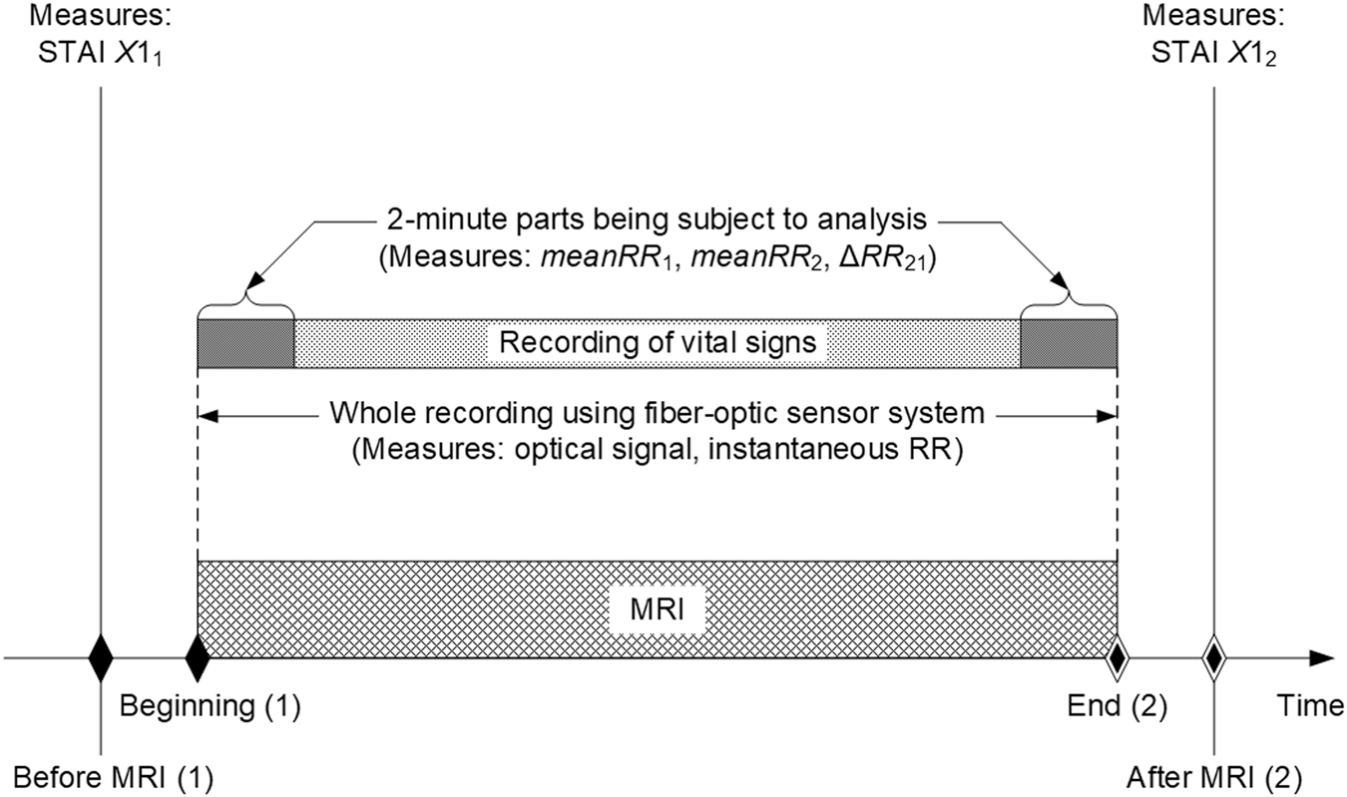
General scheme for acquiring data.

The analysis concerned changes in RR during MRI, with a particular emphasis on the beginning and the end of the examination, i.e. 2-minute parts of the whole recording, as shown in Fig. 2. Although it was originally assumed that 1-minute fragments would be analysed, 2-minute fragments were finally chosen to analyse as many subjects had fluctuations in RR just after entering the MRI scanner. It was observed that RR stabilised after approximately 1 minute. The mean RR values recorded at the beginning (*meanRR*_1_) and at the end (*meanRR*_2_) of the MRI scanning were compared relative to the STAI X-1 scores gathered before (*X*1_1_) and after (*X*1_2_) the MRI scanning, respectively. Table 1 lists information on the subjects, body parts being scanned and values of the parameters acquired during the study, including differences between the initial and ending values of the STAI scores (Δ*X*1_21_) and the mean RR measurements (Δ*RR*_21_). Additionally, the table contains comments on the course of the MRI examination and the reasons for resigning from the examination.

**Table 1:** Main information on the subjects and the results obtained during the study.

Subject			Body part being scanned	STAI			Respiratory rate			Comments
ID	Sex	Age		*X*1_1_	*X*1_2_	Δ*X*1_21_	*meanRR*_1_ (rpm*)	*meanRR*_2_ (rpm*)	Δ*RR*_21_ (rpm*)	
1	M	74	head	32	38	6	12.76	10.57	−2.19	
2	M	30	LS	34	34	0	17.06	14.20	−2.86	
3	F	46	CS	34	26	−8	17.86	17.43	−0.43	
4	M	19	knee	38	39	1	17.32	14.87	−2.45	
5	F	36	LS	34	34	0	21.51	22.20	0.69	
6	M	51	LS	31	28	−3	14.44	15.15	0.71	
7	F	57	CS	42	37	−5	18.41	12.73	−5.68	Anxiety during MRI examination
8	F	56	CS	32	33	1	20.35	20.85	0.5	
9	F	64	CS	39	31	−8	16.43	16.36	−0.07	
10	F	62	CS	38	37	−1	22.36	19.51	−2.85	
11	F	58	LS	69	45	−24	22.43	16.85	−5.58	
12	F	24	LS	36	52^†^	16	26^‡^	17^§^	−9	Strong anxiety, subject withdrew from MRI examination
13	F	74	LS	42	27	−15	12.84	12.42	−0.42	
14	F	34	LS	35	51	16	20.38	19.28	−1.1	Anxiety, subject was thinking of stopping MRI examination
15	M	81	head	38	39	1	23.13	16.62	−6.51	
16	M	50	LS	46	26	−20	27.54	27.69	0.15	Anxiety, subject took sedatives the day before MRI examination
17	F	26	CS	20	25	5	19.69	16.92	−2.77	
18	F	81	LS	62	65	3	19.69	19.79	0.1	Anxiety during MRI examination
19	M	43	CS	33	39	6	19.86	18.84	−1.02	
20	M	57	CS	37	31	−6	17.32	17.18	−0.14	
21	F	39	head**	30	23	−7	28.31	22.08	−6.23	
22	F	42	CS	26	23	−3	20.51	19.66	−0.85	
23	F	16	shoulder	32	29	−3	14.76	15.97	1.21	
24	F	67	LS	42	36	−6	21.63	18.69	−2.94	Anxiety during MRI examination
25	F	28	head**	36	33	−3	21.10	14.98	−6.12	
26	F	48	CS	34	32	−2	17.11	17.17	0.06	
27	F	43	head**	38	37	−1	16.03	16.23	0.2	
28	F	31	head	40	44	4	18.57	14.78	−3.79	
29	F	47	CS	33	53	20	18.06	20.29	2.23	Anxiety, subject was thinking of stopping MRI examination
30	F	59	knee	32	22	−10	16.05	19.32	3.27	
31	M	23	knee	35	30	−5	18.60	17.59	−1.01	
32	M	53	knee	41	41	0	22.19	22.23	0.04	
33	F	50	foot	22	27	5	20.23	21.55	1.32	Anxiety during MRI examination
34	F	41	head**	37	35	−2	19.39	15.66	−3.73	
35	F	38	LS	37	20	−17	19.05	17.00	−2.05	
36	M	60	LS	29	75^†^	46	30.18^††^	19^§^	−11,18	Panic, subject interrupted MRI examination
37	M	57	LS	30	34	4	14.44	14.77	0.33	
38	M	32	LS	35	40	5	12.85	13.28	0.43	
39	M	65	LS	21	22	1	16.55	16.59	0.04	
40	F	68	LS	39	21	−18	25.44	22.28	−3.16	
41	F	78	LS	33	30	10	15.01	15.17	0.16	
42	F	77	LS	42	44	2	15.27	16.81	1.54	
43	F	52	LS	63	48	−15	13.19	13.33	0.14	
44	F	54	LS	37	52^†^	15	28^‡^	18^§^	−10	Strong anxiety, subject withdrew from MRI examination

*Respiration per minute.

^†^Value obtained with the STAI questionnaire just after resignation or interruption of the MRI examination.

^‡^Value measured with the capnograph just after resignation from the MRI examination.

^§^Value measured with the capnograph after the subject calmed down and RR stabilised.

**Administration of contrast agents.

^††^Mean value measured with the fibre-optic sensor system within 1 minute after the MRI examination beginning.

From among 44 subjects (30 women, 14 men), one subject interrupted the examination due to hyperventilation and panic attack and two subjects withdrew from the MRI examination as a result of strong anxiety. Additionally, seven other subjects reported anxiety during the MRI procedures. This in turn gives 6.8%-level of withdrawals and 22.7%-level of declared anxiety symptoms. The percentage values obtained in our study are within the range of the literature data highlighted previously. Based on this classifications, Firth’s logistic regression models for predicting withdrawal (3 cases) and reported anxiety (10 cases) were estimated with the *meanRR*_1_ values and STAI *X*1_1_ scores as predictor variables. These both predictors were centred around the group mean before entering into regression equations. With *meanRR*_1_, both models for withdrawal and for anxiety reporting were significant (likelihood tests with *p* < 0.001 and *p* = 0.003, respectively). In the assessed group, for subjects with an average *meanRR*_1_ value there was a 1%-probability of withdrawal, and for a unit change in *meanRR*_1_, odds for withdrawal were expected to increase by a factor of 1.66 (with 95% CI from 1.20 to 3.51). The probability of anxiety reporting among subjects with an average *meanRR*_1_ value was approximately 23%, and odds for a unit change in *meanRR*_1_ were expected to increase by a factor of 1.29 (95% CI from 1.09 to 1.60). To assess the goodness of fit of the estimated models, Tjur’s coefficient of discrimination^21^ was computed with the values of 0.48 for withdrawal and 0.47 for anxiety reporting, showing moderate explanatory power for both models. With *X*1_1_, neither the model for withdrawal nor model for anxiety reporting showed statistical significance (likelihood tests with *p* = 0.122 and *p* = 0.939, respectively).

In the second step, data obtained from 41 MRI patients who completed their scans (28 women and 13 men, aged 50.1 ± 17.4 years) were used for statistical analyses. The data are provided in a CSV file as the supplementary material. At first, exploratory data analysis was performed. Descriptive statistics for repeated measures, as well as differences and correlations of the measurements collected using the STAI questionnaire before and after the MRI examination, and using the fibre-optic sensor system at the beginning and at the end of the MRI examination, are presented in Table 2. The mean difference between anxiety state obtained after and before the MRI examination is M = −2.56 (SD = 8.77). Although the overall change is not significant, substantial variability among individuals is observed. Almost all variables are approximately normally distributed, as confirmed by graphical inspection of the distributions and with the Shapiro-Wilk test. Only the first measurement of anxiety state is slightly right-skewed.

**Table 2:** Results of anxiety and RR measurement at the beginning and end of the MRI study (N = 41).

*X1*_*1*_		*X1*_*2*_		*ΔX1*_*21*_		*t*	*r*	Cohen’s d
M	SD	M	SD	M	SD			
36.93	9.7	34.37	9.55	−2.56	8.77	1.869	0.59^†^	0.294
***meanRR***_***1***_		***meanRR***_***2***_		***ΔRR***_***21***_		***t***	***r***	**Cohen’s d**
**M**	**SD**	**M**	**SD**	**M**	**SD**			
18.68	3.73	17.44	3.33	−1.24	2.41	3.295*	0.77^†^	0.936

*p < 0.01; ^†^*p* < 0.001.

There is no significant correlation between the STAI *X*1_1_ score and the *meanRR*_1_ from the onset of MRI examination (*r*_(39)_ = 0.08, *p* = 0.626, 95% CI [−0.23; 0.038]). It can be concluded that there is no visible relationship between initial anxiety level and initial RR.

**Figure 3:**
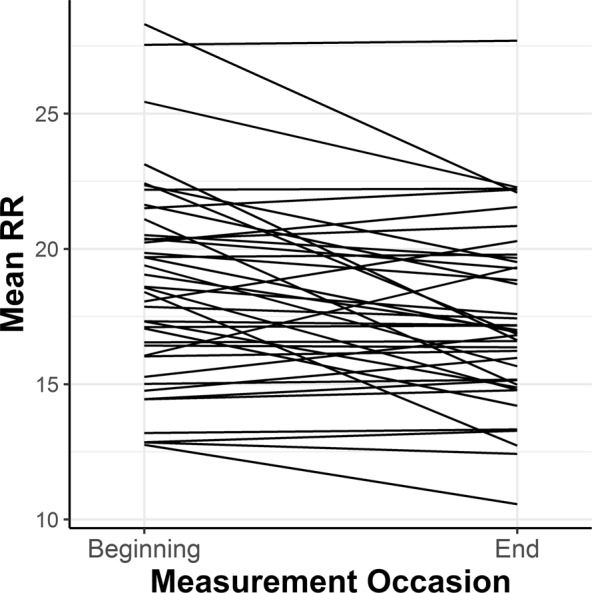
Spaghetti plot of individual changes in the mean RR among study participants.

**Figure 4:**
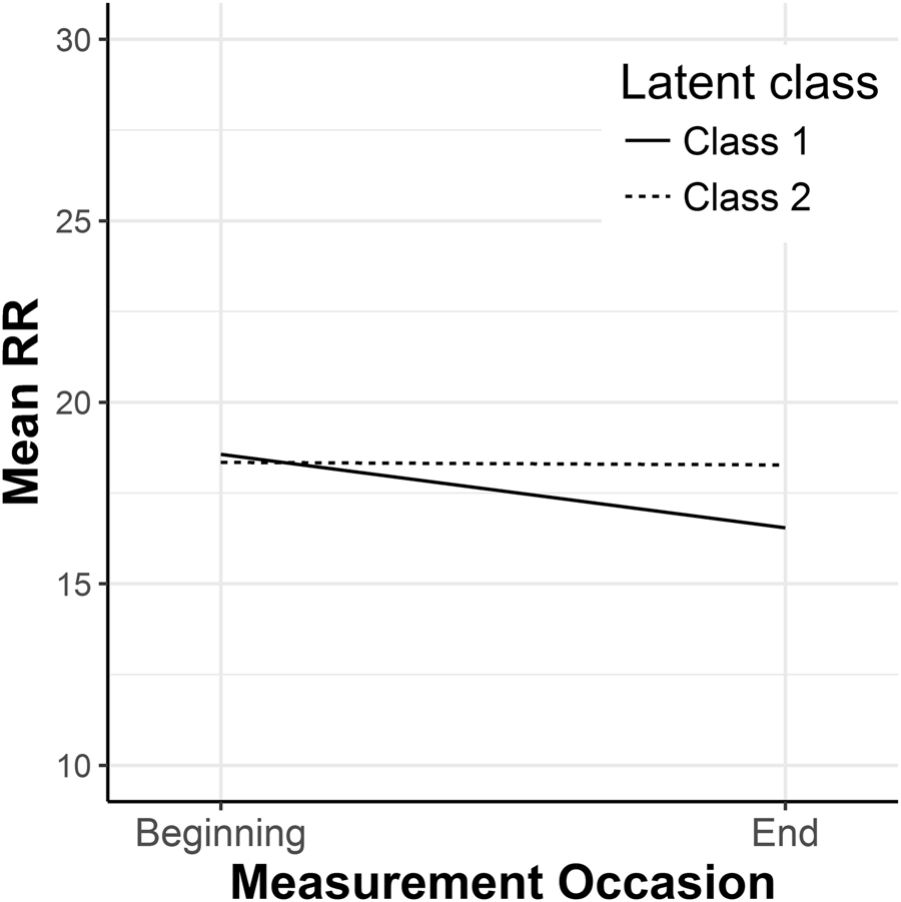
Class-specific mean predicted trajectories for the two latent classes from the growth mixture model analysis.

In addition to a significant decrease of the mean RR from the initial level to the ending level, Δ*RR*_21_, there was considerable individual variability in the trend among all participants, as shown in Fig. 3. Therefore, a growth mixture model analysis was applied to extract groups of subjects with homogenous change trajectories. Measurement occasions were included as an ordered factor and allowed to vary across possible latent classes. The intercept was excluded from the mixture equation, thus only the magnitude of change, regardless of *meanRR*_1_, was taken into consideration while searching for latent classes. Model fitting procedures for the two-class mixture model resulted in a log likelihood value of −198.94 and a Bayesian information criterion (BIC) of 431.3. The estimate for the intercept was significant (*M*_i_ = 18.345, *p* < 0.001) with the variance of the random effect at 6.84. For Class 1, the estimate of RR change during measurement was *M*_c_ = −2.031, *p* = 0.038, while for Class 2, the estimate of change value was not significant, *M*_c_ = −0.071, *p* = 0.901. The reproduced means for the two-class model are shown in Fig. 4. A significant decrease in the mean RR from the initial level to the ending level, Δ*RR*_21_, is observed in Class 1, whereas Class 2 is distinguished by having almost the same level of RR at the beginning and at the end of the MRI examination. Means of posterior probabilities in each class were 0.851 and 0.149 in Class 1 and 0.301 and 0.699 in Class 2. Classification of individuals based on their most likely class membership resulted in class counts and proportions of 19 (46.34%) in Class 1 and 22 (53.66%) in Class 2.

Given the significant estimates derived from the two-class model, the viability of a three-class solution was examined. The model fitting procedures for the three-class mixture model resulted in a worse model fit based on a BIC (*BIC* = 442.44). The third latent class appeared to be a split of Class 2 from the two-class mixture model, with a non-significant trend in the change in mean RR. Therefore, the three-class model was rejected as a model with no additional explanatory value for estimating the patterns of change. Thus, the two-class model classification was accepted for further analysis.

In the next step, the two classes were assessed for between-group differences. After checking for normal distributions and homogenous variances, the one-sided Student’s *t*-test was performed on the changes in anxiety state. A sensitivity power analysis showed that with the available sample size, *alpha* = 0.05 and *power* = 0.80, while the minimal detectable effect is approximately Cohen’s *d* = 0.79.

**Figure 5:**
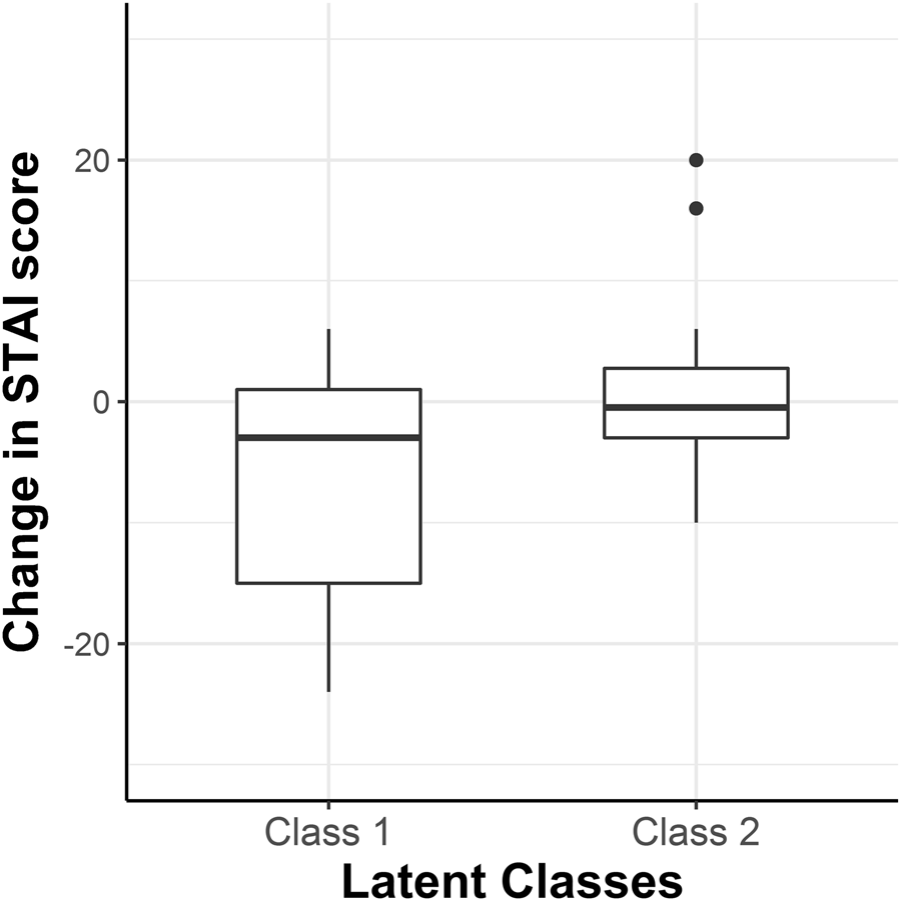
Magnitude of changes in anxiety state in the two latent classes (scores above 0 mean an increase in the STAI scores from the first to the second measurement).

The results of the *t*-test showed significant differences (*t*_(39)_ = −2.349, *p* = 0.012, Cohen’s *d* = 2.13) in changes in anxiety between the groups, with a mean change value of −5.84 (*SD* = 9.48) in Class 1 and 0.27 (*SD* = 7.16) in Class 2, as shown in Fig. 5. The effect size of the detected difference could be classified as a large effect, showing that there was a significant lowering of anxiety state in the group characterised by the decrease in RR, and practically no change in anxiety level in the group with a stable (non-decreasing) RR.

In addition to the results of statistical analysis, valuable information is provided by the data derived from three subjects not included in the statistical analysis. One of the subjects (#36) interrupted the MRI examination one minute after its beginning. At that time, the mean RR was 30 rpm, while breathing became shallower; the subject experienced a state typical of hyperventilation. The STAI questionnaire survey showed an increase in the X-1 subscale from the level determined on the basis of the sten scale^15^ as a “low” intensity of anxiety symptoms before the examination to the “high” level after its interruption. This increase was by as much as 46 points and it was the highest value obtained from the subjects. The subject calmed down 15 minutes after the examination interruption; his RR decreased and stabilised at the level of approximately 19 rpm, when measured using the capnograph. Two subjects who resigned from the MRI examination were characterised by an accelerated RR, i.e. 26 rpm (#12) and 28 rpm (#44), when measured with the capnograph immediately after their resignation. In both cases, an increase in the STAI X-1 subscale from the “medium” level before the MRI to the “high” level after resigning from the scan in terms of the intensity of anxiety symptoms was observed. After 10 minutes, RR decreased and stabilised at the level of 17 rpm and 18 rpm for subject #12 and subject #44, respectively.

A similar feeling in terms of increasing level of anxiety was experienced by subjects #14 and #29, who considered interrupting the MRI examination during its course. Like patients #12 and #44, they declared in the STAI questionnaire an increase in the level of anxiety from “medium” before the scanning to “high” after its completion, with the difference being that their maximum RR was only slightly above 20 rpm. It can be hypothesised that the same level of anxiety in some subjects causes a considerable increase in RR while in others it does not. On the other hand, the STAI questionnaire allows for a subjective assessment of the intensity of anxiety symptoms. Thus the actual level of anxiety may have been lower in subjects who undertook the MRI examination and, against their feelings, did not decide to interrupt it.

Another interesting case was represented by subject #16, who knew his claustrophobic tendencies and took sedatives the day before the study. It was his second attempt to undertake the MRI examination, as he resigned from the examination the first time due to strong anxiety and hyperventilation. The subject was under the influence of sedatives and was clearly subdued; he talked to himself during the MRI examination and claimed that this calmed him down. The completion of the MRI examination brought noticeable relief to the subject, which was reflected in the decrease in the STAI X-1 subscale from the “high” level before scanning to “low” level after finishing the examination. At the same time, the RR value remained practically unchanged throughout the entire examination and was approximately 27.5 rpm.

An alarming value of RR, as high as 28 rpm, at the beginning of the MRI examination could be observed in subject #21. However, during the course of the examination, this value constantly decreased and achieved 22 rpm by the end of the scanning. The subject was aware of the accelerated and irregular breathing and, as she explained after the examination, she tried to calm down and stabilise her breathing. In the STAI questionnaire, the subject declared “low” levels of anxiety both before and after the MRI examination. This lack of anxiety in the subject may have been positively influenced by coping with the increased RR using her own techniques.

The above-described individual cases of the distinctive subjects show that their RR indicator was mostly compatible with their current psychophysiological state and well-being in the study situation. All the patients who were afraid of the MRI examination and decided to resign from the scanning distinguished with high RR values, i.e. above 25 rpm, at the first measurement. In the patients who declared anxiety and/or were thinking of stopping the examination, at least slightly elevated RR values at the beginning of the scanning were observed. These regularities justify the need for continuous monitoring RR in MRI patients, which can be realised by minimal means using easily-implementable fibre-optic sensors.

## Discussion

One of the most important findings of the study is the ability to predict, at least to some extent, the possibility of anxiety incidents during MRI, based solely on the RR measurement. While the declared level of psychological tension, measured by the STAI questionnaire at the beginning of the MRI procedures, was not clearly connected with the possibility of resignation from the examination, highly increased RR appeared to be a good predictor of subject’s increased anxiety, or even withdrawal from the examination. This result legitimises the RR measurement during MRI and, on the other hand, shows that the declared, subjective level of anxiety does not always translate into behaviour. It is possible that the relationship between the initial level of anxiety and the actual behaviour is not straightforward, and there could be involved some coping mechanism in it. Presumably, some people are more able than others to self-control and reduce their anxiety level in difficult and stressful situations, however such a factor was not controlled during this study.

Although normative information regarding subjective anxiety, i.e. inter-subject comparisons and assessment of results as low/high against the average group scores, are not directly related to RR during the MRI examination, the within-subject relative changes in the declared anxiety are clearly connected with the changes within RR, as showed in the second part of the statistical analysis. In the group of subjects distinguished by a lowering of RR during MRI scan, there was a relative reduction of subjectively perceived anxiety, while in the group with a stable or slightly increasing RR, the declared anxiety level was similar before entering and after leaving the MRI scanner. These observations are of fundamental meaning if the patient’s anxiety control is to be implemented in the medical procedure, e.g. to minimise the number of withdrawals. They suggest that after assessing a patient’s initial anxiety level using an interview, self-reporting tools, etc., further monitoring of RR during the course of the MRI scan can be a sufficient and non-invasive method to control the level of emotional tension experienced by the patient. As observed in the study, in the situation in which a subject declares a clearly elevated level of anxiety before the examination, a lack of slowing down the RR during the scan may be a symptom of persistently high emotional tension. Hence, this could be treated as a signal for the MRI operator to take some actions in order to minimise the risk of withdrawal. On the other hand, in a situation in which the initial self-assessed anxiety is not high, even a relatively high level of RR throughout the entire examination does not necessarily mean that there is a significant risk that the patient will decide to prematurely stop the examination. Among patients in whom a reduction in RR can be observed, one can also expect a decrease in the subjective anxiety, regardless of its initial level.

The results of the statistical analyses as well as the descriptions of individual cases that occurred during our study demonstrate that respiratory monitoring provides valuable information to the MRI operator and should be implemented in every scanning. At the beginning of an MRI examination, the operator can identify patients with an increased and irregular RR for monitoring of possible claustrophobic behaviours. The study shows that patients breathing at an RR above 20 rpm should be taken into consideration, and those with an RR more than 25 rpm require particular attention. By observing the trends in RR changes, the operator can predict the behaviour of the patient. Decreasing trends in RR changes suggest that the patient will complete the MRI without incident. The persistence of a high level of RR, often of an irregular nature, may lead to hyperventilation and panic attack. Early detection of anxiety-related symptoms enables the operator to take appropriate actions in order to avoid panic attack or even psychological discomfort in a patient. A properly trained operator can then stop the scanner, calm the patient down with a relaxing conversation or breath control techniques, e.g. deep breathing exercises, and return to the examination after a short break. The literature shows that MRI anxiety can be effectively reduced even by only providing patients with standard information about the course of the examination and the scans to be made^22,23^. In more complex cases, in which the level of anxiety is too high and the patient cannot calm down quickly, the operator may recommend the patient first undergo some relaxation training to reduce hyperventilation and anxiety effects and, afterwards, make the next attempt to undertake and successfully complete MRI examination^24,25^.

Relaxation training and techniques, aimed at psychophysical relaxation, support conventional methods of pharmacological treatment without causing adverse reactions or generating additional high costs. Breathing control, behavioural relaxation training and biofeedback are beneficial to reduce anxiety, relax an increased muscle tone, normalise elevated blood pressure and accelerated heart and respiratory rates, as well as to improve subjective control of perceived stress^26^. One of the studies revealed relaxation techniques that minimised the state of emotional activation and generated a state of emotional containment during the waiting period before diagnostic screening in cancer patients^27^. Considering the above, in cases in which MRI patients suffer from excessive anxiety and tension and cannot finish the examination, it is recommended that they be informed of the possibility of undergoing different relaxation trainings that teach how to effectively deal with negative states, i.e. tension, anxiety and stress, and, as consequence, reach a state of calm and mental relief.

Research conducted in this area also shows that listening to music during MRI scanning significantly reduces patients’ anxiety and makes the examination itself more pleasant. It was found that commonly used noise-cancellation devices, i.e. earplugs and headphones, did not cause the same effect as music^28^. Therefore, turning on music for MRI patients, including relaxing tones, can be an effective, inexpensive, fast and safe way to reduce tension and stress caused by the examination situation. Moreover, the literature shows that using audio-visual systems reduces patient motion and leads to a quality diagnostic MRI without the use of sedation^29^. Other authors confirm this thesis in their study on auditory and olfactory stimulation during MRI scanning^30,31^. Sensory stimulation in the form of calming bird noises played over headphones and a scented cotton pad placed in the scanner near the patients’ heads has improved the overall subjective experience of MRI in the patients. These low-cost interventions are well tolerated by patients and can be particularly helpful in those with high anxiety to improve their well-being and increase the possibility of completing the examination in the MRI chamber.

The results and interpretations have their limitations. One of them is associated with a moderate sample size, which resulted in only a few cases with a significant increase in RR and/or increase in declared anxiety level. Because of this range restriction in the observed data, it was not possible to statistically assess to what extent an increase in RR may be a predictor of an increase in anxiety, even in patients who did not declare excessive emotional tension in the initial phase. The above-mentioned observation can only be treated as qualitative and based on incidental results. Access to data collected on a larger, more diverse group will give a better estimate of this potential dependence, and this is planned as future work.

Another limitation is associated with the method of measuring anxiety. Due to the research possibilities, the declared level of anxiety was assessed in a standardised manner, before and after the MRI examination, without controlling its level during the examination itself. This reduces the analysis of changes in anxiety level to a linear trend only and is, indeed, a weakness of the questionnaire method, as was mentioned in the introduction. However, a careful inspection of changes in RR throughout the whole examination indicates that a general trend is accurately expressed by the difference between the final and initial level of RR and subject to visible fluctuations during the course of the MRI procedures. If the measurement of the declared level of anxiety is realised in a relatively non-intrusive manner at several time points during the MRI examination, a more precise picture of the relationship between the subjectively assessed emotional state and observable physiological variables can be obtained. Such a study will require developing a method that will allow a subjective assessment of perceived anxiety with a standardised indicator. The easiest way to achieve this is a cyclic request to the subject to assess his/her psychological condition on a several-point scale. Other authors measured the level of anxiety using the STAI questionnaire through a series of questions asked to patients by the operator during MRI examinations^32^. However, this might influence the measurement result by continuously forcing the subject to self-monitor and reflect on his/her own emotional state. To facilitate the patient’s self-assessment, the displaying of queries on an MRI-compatible monitor screen and the use of response pads, as during a functional MRI (fMRI) study, may be considered^33^.

**Figure 6:**
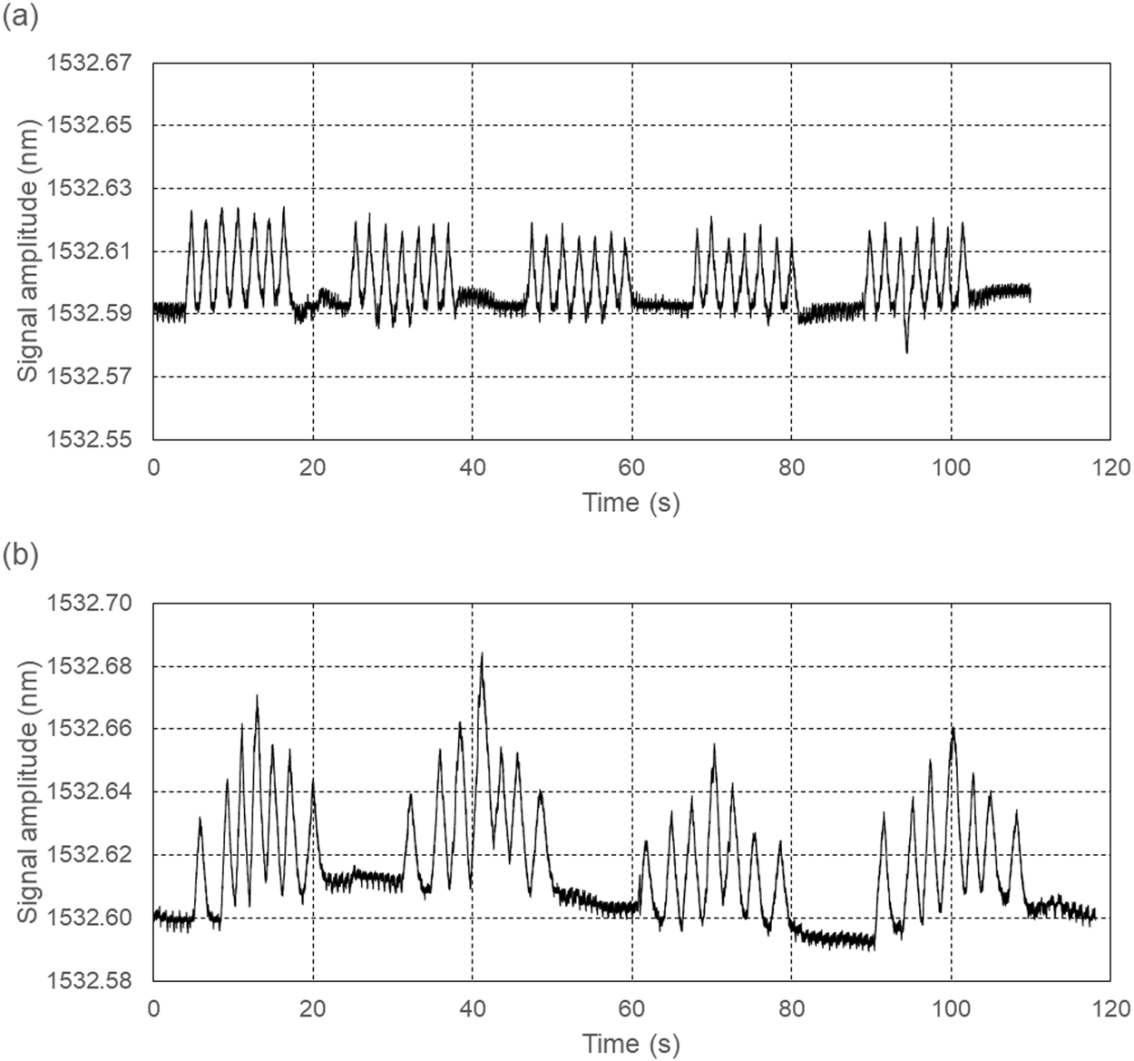
Examples of recordings of abnormal respiration using the fibre-optic sensor system: (**a**) Biot-type respiration and (**b**) Cheyne-Stokes-type respiration.

The presented analysis does not take into account the fact that an increase in RR may result not only from an elevated level of anxiety, but also from diseases that cause breathing disorders. The subjects were not asked about this type of diseases in the questionnaire. Moreover, the respiration amplitude has not been analysed so far, and as emphasised in the introduction, the depth of respiration along with its rate may also indicate the emotional state of the subject. As shown in Fig. 6, the fibre-optic sensor system perfectly copes with the recording of the respiratory curve, including its amplitude. Figure 6(a) shows an example of the recording of periodically rapid and shallow breathing interrupted by a few seconds pauses, occurring during meningitis and cerebral haemorrhages^34^. The recording shown in Fig. 6(b) is an example of irregular breathing, in which first there are slow and shallow breaths, and then more frequent and deeper breaths. When the depth and rate reach the highest degree, the breath becomes shallower and rarer until there is an apnoea. This type of breathing expresses a dysfunction of the respiratory centre in the medulla and occurs in patients with heart failure, chronic kidney disease, renal tumours or severe poisoning^35^. Connections of breathing depth and frequency with disorders in patients diagnosed with MRI will be the subject of future research.

In conclusion, the system presented here for non-invasive RR monitoring allows the MRI operator to better control the patient’s condition and to decide whether to continue the examination after its suspension or to completely stop the examination and recommend a therapy to the patient prior to completing the MRI.

## Supplementary information

Dataset 1
